# H_2_O_2_-Mediated Oxidative Stress Enhances Cystathionine γ-Lyase-Derived H_2_S Synthesis via a Sulfenic Acid Intermediate

**DOI:** 10.3390/antiox10091488

**Published:** 2021-09-18

**Authors:** Jun Wang, Guanya Jia, Heng Li, Shasha Yan, Jing Qian, Xin Guo, Ge Li, Haizhen Qi, Zhilong Zhu, Yanjun Wu, Weijuan He, Weining Niu

**Affiliations:** School of Life Sciences, Northwestern Polytechnical University, Xi’an 710072, China; junw@mail.nwpu.edu.cn (J.W.); jiagy@mail.nwpu.edu.cn (G.J.); aheng@mail.nwpu.edu.cn (H.L.); yanshasha@mail.nwpu.edu.cn (S.Y.); qianjing@mail.nwpu.edu.cn (J.Q.); 1291809650@mail.nwpu.edu.cn (X.G.); lige0815@mail.nwpu.edu.cn (G.L.); qihaizhen@mail.nwpu.edu.cn (H.Q.); zhilongzhu_2019@mail.nwpu.edu.cn (Z.Z.); wuyanjun@mail.nwpu.edu.cn (Y.W.); 2514997391@mail.nwpu.edu.cn (W.H.)

**Keywords:** cystathionine *γ*-lyase, hydrogen sulfide, hydrogen peroxide, sulfenic acid modification, oxidative stress

## Abstract

Hydrogen sulfide (H_2_S), which is generated mainly by cystathionine *γ*-lyase (CSE) in the cardiovascular system, plays a pivotal role in a wide range of physiological and pathological processes. However, the regulatory mechanism of the CSE/H_2_S system is poorly understood. Herein, we show that oxidation induces the disulfide bond formation between Cys252 and Cys255 in the CXXC motif, thus stimulating the H_2_S-producing activity of CSE. The activity of oxidized CSE is approximately 2.5 fold greater than that of the reduced enzyme. Molecular dynamics and molecular docking suggest that the disulfide bond formation induces the conformational change in the active site of CSE and consequently increases the affinity of the enzyme for the substrate L-cysteine. Mass spectrometry and mutagenesis studies further established that the residue Cys255 is crucial for oxidation sensing. Oxidative stress-mediated sulfenylation of Cys255 leads to a sulfenic acid intermediate that spontaneously forms an intramolecular disulfide bond with the vicinal thiol group of Cys252. Moreover, we demonstrate that exogenous hydrogen peroxide (H_2_O_2_) and endogenous H_2_O_2_ triggered by vascular endothelial growth factor (VEGF) promote cellular H_2_S production through the enhancement of CSE activity under oxidative stress conditions. By contrast, incubation with H_2_O_2_ or VEGF did not significantly enhance cellular H_2_S production in the presence of PEG-catalase, an enzymatic cell-permeable H_2_O_2_ scavenger with high H_2_O_2_ specificity. Taken together, we report a new posttranslational modification of CSE that provides a molecular mechanism for H_2_O_2_/H_2_S crosstalk in cells under oxidative stress.

## 1. Introduction

Hydrogen sulfide (H_2_S) is an endogenous signaling molecule that mediates diverse biological functions. In mammalian cells, H_2_S is produced enzymatically by cystathionine *β*-synthase (CBS), cystathionine *γ*-lyase (CSE) and 3-mercaptopyruvate sulfur transferase (3-MST) [1,2], among which CSE is the predominant H_2_S-generating enzyme in the cardiovascular system, specifically in vascular endothelial cells, smooth muscle cells, and cardiomyocytes [3,4]. Growing evidence indicates that a wide range of physiological and pathological processes can be mediated by H_2_S in the cardiovascular system, including blood vessel relaxation, cardioprotection, and atherosclerosis [5,6,7]. Despite the major importance of CSE-derived H_2_S production, the regulatory mechanism of the CSE/H_2_S system is less well understood.

CSE belongs to the γ-family of pyridoxal 5′-phosphate (PLP)-dependent enzymes and plays a central role in the trans-sulfuration pathway. CSE can catalyze the cleavage reaction of L-cystathionine to cysteine, the limiting substrate in the synthesis of the antioxidant glutathione. Alternatively, CSE can catalyze the condensation of two molecules of cysteine to produce H_2_S [8,9]. Therefore, enzymes in the trans-sulfuration pathway play a key role in maintaining the cellular redox homeostasis, and the activity of the rate-limiting enzymes could be reversibly regulated in response to redox changes. Indeed, previous studies reported that exposure of human umbilical vein endothelial cells (HUVECs) to H_2_O_2_ or vascular endothelial growth factor (VEGF) stimulates the cellular release of H_2_S, whereas no significant difference was observed in the expression level of CSE, implying that the H_2_S-producing activity of CSE should be posttranslationally regulated [10,11]. However, the molecular mechanism underlying the redox regulation of the CSE/H_2_S system is very poorly understood.

CSE is a homotetramer of approximately 45 kDa subunits and contains two highly conserved CXXC motifs, C252YLC255 and C307TGC310, which exist in reduced states in the crystal structure (PDB: 2NMP) [9]. Interestingly, these two CXXC motifs begin to appear in vertebrates, suggesting that these two CXXC motifs may have specific biological functions in sensing the redox state of the cellular environment. We hypothesized that the formation of the disulfide bond in one of the two CXXC motifs in CSE might stimulate enzyme activity to concomitantly enhance CSE-derived H_2_S production in cells exposed to stress conditions. The role of the CXXC motif may act as a redox switch in response to oxidative stress. In the present study, we demonstrated that a redox-sensitive cysteine residue (Cys255) of CSE can form an intramolecular disulfide bond with the vicinal cysteine residue (Cys252) in the C252XXC255 motif. The C252XXC255 motif in CSE exists in reduced and oxidized states in recombinant protein and in human aortic vascular smooth muscle cells (HA-VSMCs). The activity of oxidized CSE increased by approximately 2.5-fold compared with reduced CSE and resulted in a concomitant increase in CSE-derived H_2_S in response to hydrogen peroxide (H_2_O_2_)-induced oxidative stress or VEGF treatment in HA-VSMCs. This discovery showcases an intrinsic oxidation-sensing mechanism of the CSE/H_2_S system that has broad implications for understanding the physiological roles of H_2_S, especially in the cardiovascular system.

## 2. Materials and Methods

### 2.1. Materials

The anti-cystathionine *γ*-lyase (anti-CSE) antibody and the anti-cystathionine *β*-synthase (CBS) antibody were obtained from ABclonal (Wuhan, China). The fluorescent thiol probe 7-diethylamino-3-(4-maleimidophenyl)-4-methylcoumarin (CPM) and *S*-methylcysteine were purchased from Sigma-Aldrich (St. Louis, MO, USA). The fluorescent probe SF7-AM for H_2_S detection was obtained from Cayman (Ann Arbor, MI, USA). The methyl-PEG-maleimide reagent MM[PEG]_24_ was purchased from AAT Bioquest (Sunnyvale, CA, USA). The cell counting kit-8 was purchased from Dojindo (Kumamoto, Japan). Unless otherwise specified, all chemicals were used as received.

### 2.2. Protein Expression and Purification

Mutagenesis of human CSE was carried out using a Quikchange II XL Site-directed Mutagenesis Kit (Agilent Technologies, Santa Clara, CA, USA). The mutations were verified by DNA sequencing (Sangon, Shanghai, China). The expression and purification of the wild-type and variant versions of CSE were performed as described previously [12]. The purified protein was stored at −80 °C and the purity was assayed by SDS-PAGE analysis.

### 2.3. H_2_S Production Assays

Generation of H_2_S by CSE was measured using L-cysteine as the substrate in a spectrophotometric assay described previously [13,14,15]. The reaction of H_2_S production by CSE with lead acetate to form lead sulfide was monitored at 390 nm. After the reaction solution (20 mM L-cysteine, 0.4 mM lead nitrate, 50 mM Hepes buffer, pH 7.4) was preincubated at 37 °C for 5 min, the reaction was initiated by the addition of 10 µg CSE and was monitored in a multifunctional microplate reader at 37 °C for 10 min. Reduced CSE and oxidized CSE were prepared as follows. Recombinant CSE was incubated with 10 mM dithiothreitol (DTT), 5 mM oxidized dithiothreitol (DTT_oxi_, trans-4,5-dihydroxy-1,2-dithiane; Sigma) or 400 µM H_2_O_2_ for 1 h at room temperature. DTT_oxi_ was used to induce the disulfide bond formation through the thiol-disulfide exchange reaction. H_2_O_2_ was employed to oxidize the redox-active thiols in CSE to sulfenic acid intermediate that could spontaneously form a disulfide bond with a neighboring thiol group. The reduced CSE was reoxidized by H_2_O_2_ or by exposing it to air oxygen for 1 h at room temperature by gently pipetting up and down. The samples were then ultrafiltered to remove DTT, DTT_oxi_ or H_2_O_2_. The concentration of each protein sample was determined using a BCA protein assay kit (TransGen Biotech, Beijing, China). An extinction coefficient of 5500 M^−1^ cm^−1^ at 390 nm was used for estimation of the lead sulfide concentration.

### 2.4. Measurement of CSE Activity Using S-Methylcysteine as a Substrate

The CSE activity using *S*-methylcysteine, a thiol-free substrate analog of cysteine, as a substrate was determined according to our previously reported method with some modifications [12]. CSE can catalyze the decomposition of the *S*-methylcysteine to produce methanethiol (CH_3_SH), which can be measured by the fluorescent thiol probe CPM (7-diethylamino-3-(4-maleimidophenyl)-4-methylcoumarin, Sigma). The reaction mixture containing 50 mM Hepes buffer (pH 7.4), 10 mM *S*-methylcysteine was added to a 96-well plate, and the reaction was started by adding 10 µg of CSE. After the reaction was carried out for 10 min at 37 °C, the fluorescent thiol probe CPM (15 µM) was added to the reaction mixture. The fluorescence of the reaction mixture was detected at 460 nm (λex = 400 nm) by a multifunctional microplate reader. The reaction mixture without CSE protein was set as a blank control. The activity of oxidized CSE was not affected by 100 µM sodium thiomethoxide (CH_3_SNa) treatment for 10 min at 37 °C (Figure S1). Alternatively, DTNB (5,5′-dithiobis-2-nitrobenzoic acid) was used to detect production of CH_3_SH using an extinction coefficient of 13,600 M^−1^cm^−1^ at 412 nm for 2-nitro-5-thiobenzoic acid anion [13,16].

### 2.5. Mass Spectrometry

A solution of CSE was ultrafiltered and buffer-exchanged using a Microcon YM-10 centrifugal filter device (Millipore) with 50 mM ammonium bicarbonate. The free sulfhydryl groups of the samples were blocked by alkylation with 25 mM iodoacetamide in the dark for 1 h. After buffer exchange against 50 mM ammonium bicarbonate, trypsin or chymotrypsin was added into the protein solution at 37 °C for 16–18 h. The digested peptides were extracted and lyophilized. Subsequently, the peptides were redissolved in 20 μL of 0.1% formic acid for LC-MS/MS analysis. All samples were analyzed utilizing a Thermo Ultimate 3000 Nano HPLC system equipped with a Reprosil-Pur C18-AQ column (Dr. Maisch GmbH, Ammerbuch-Entringen, Germany). The mobile phases consisted of A (0.1% (*v*/*v*) formic acid in H_2_O) and B (0.1% (*v*/*v*) formic acid in 80% (*v*/*v*) acetonitrile) at a flow rate of 0.3 μL/min. The gradient elution was performed as follows: 0–5 min, 6–9% B; 5–20 min, 9–14% B; 20–50 min, 14–30% B; 50–58 min, 30–40% B; 58–60 min, 40–95% B. An HPLC system coupled to a Q Exactive hybrid quadrupole-Orbitrap mass spectrometer (Thermo Finnigan) was used to detect the peptides. The mass spectrometer was operated in positive electrospray ionization mode using a data-dependent acquisition.

A survey scan (*m*/*z* range, 300–1800) was acquired at a resolution 70,000 at *m/z* 200, with an accumulation target value of 3,000,000 ions. The 20 most intense ions were sequentially isolated and fragmented using collisionally induced dissociation with an activation q value at 0.25 within the linear ion trap. Former target ions selected for MS/MS were dynamically excluded for 30 s. Mass spectra and collision MS/MS data were searched against the equivalent theoretical masses from the National Center for Biotechnology and Information database. The search parameters were as follows: precursor ion mass tolerance 20 ppm, and fragment ion mass tolerance ± 0.02 Da. Methionine oxidation, protein N-term acetylation, and carbamidomethylation of cysteine were selected as variable modifications.

In the case of determination of the sulfenic acid modification, the wild type CSE and C252S mutant protein solutions were exposed to air oxygen, pipetting up and down six to eight times, and incubated for 1 h at room temperature. Subsequently, the samples were incubated with dimedone (5 mM) for sulfenic acid labeling. Finally, the sulfenic acid modification site was detected using liquid chromatography tandem mass spectrometry (LC-MS/MS) as described above. Dimedone labeling, carbamidomethylation of cysteines, methionine oxidation, and protein *N*-term acetylation were selected as variable modifications.

### 2.6. Labeling of Unpaired Cysteine Thiols in CSE

The unpaired cysteine thiols of CSE were labeled according to previously reported methods with some modifications [17,18]. The human aorta vascular smooth muscle cells (HA-VSMCs) were cultured in DMEM supplemented with 10% (*v*/*v*) fetal bovine serum at 37 °C and 5% (*v*/*v*) CO_2_. When approximately 80% confluence was reached in a 10-cm plate, DTT or H_2_O_2_ was added to a final concentration of 400 μM. After an incubation of 30 min, the cells were washed three times with ice-cold PBS and then lysed with 500 μL RIPA buffer (Solarbio, Beijing, China). Subsequently, the cell lysates were incubated with 10% (*v*/*v*) TCA on ice for 30 min. The precipitate was washed three times with cold acetone followed by a final wash with 75% ethanol. Subsequently, the precipitate was redissolved in 100 μL of phosphate saline buffer containing 3% (*w/v*) SDS, pH 7.0, followed by the addition of MM[PEG]_24_ at a final concentration of 20 mM. After an overnight incubation at room temperature, the labeled samples were resolved by SDS-PAGE followed by Western blot analysis with CSE antibody (A6121, ABclonal, Wuhan, China). The molecular mass of MM[PEG]_24_ is 1240 Da.

### 2.7. Determination of Unpaired Cysteine Thiols in CSE

The CSE protein was precipitated by 10% (*v*/*v*) TCA and redissolved in phosphate buffer containing 3% (*w*/*v*) SDS, pH 7.0 as described above. Then, unpaired cysteine thiols in CSE were determined using a Total Thiol Assay Kit (Solarbio, Beijing, China) based on the reaction with DTNB according to the Ellman method. The protein concentration was determined using a BCA Protein Assay Kit (TransGen Biotech, Beijing, China).

### 2.8. Molecular Dynamics (MD) Simulations of the Reduced CSE and the Oxidized CSE

Crystal structure of the tetrameric CSE in the reduced form (PDB ID: 2NMP) was used as the initial structure for molecular dynamics simulations [9]. Two systems were set up and designated as Reduced-CSE and Oxidized-CSE. To model the initial structure of Oxidized-CSE, a disulfide bond was created between the two S atoms of Cys252 and Cys 255, which were separated by 7.1 Å in the reduced CSE crystal structure. Then, the residues within 5 Å of Cys252 and Cys255 were subjected to energy minimization with an amber force field by using the following parameters: a distance-dependent dielectric function, nonbonded cutoff of 8 Å, amber charges for the proteins, whereas other residues were treated with position restraint using a force constant of 1000 kcal/mol/Å^2^. The resulting systems were first minimized for 2000 steps by the steepest descent method followed by the conjugate gradient method for 2000 steps.

All MD simulations were performed using the GROMACS 2018.4 package with constant temperature, pressure and periodic boundary conditions [19]. The Amber99SB protein force field and TIP3P water model were employed [20,21,22,23]. In the MD simulations, the Particle-Mesh Ewald (PME) method was utilized to address the electrostatic interactions, and a nonbonded cutoff of 10 Å was applied [24]. The nonbonded pairs were updated every 10 steps. All chemical bonds involving hydrogen atoms were constrained using the LINCS algorithm, and the time step used for the MD simulations was 2 fs. The MD simulations were run at a constant pressure of 1.0 bar, by applying the Parrinello-Rahman barostat, at 300 K using the Berendsen thermostat [25,26]. First, the steepest descent method was employed to minimize the energy of the two systems and remove unfavorable contacts. Subsequently, the system was gradually heated from 0 to 300 K over a period of 250 ps and then equilibrated for 250 ps. Finally, a 100-ns MD simulation was conducted for each system, with coordinates saved every 20 ps.

### 2.9. Molecular Docking

Molecular docking of l-cysteine with Reduced-CSE and Oxidized-CSE was carried out using the AutoDock 4.2.6 program (The Scripps Research Institute, La Jolla, CA, USA). The initial structure was retrieved from the MD simulation and subjected to energy minimization. The structure of l-cysteine was downloaded from the PubChem database, and then the MOPAC program was used to optimize the structure and calculate the PM3 atomic charges [27,28]. The structures of both receptors and ligand were prepared by using AutoDock Tools 1.5.6, and the corresponding pdbqt files were generated for receptor-ligand molecular docking [29]. The active site of CSE was chosen as the binding pocket for docking. The number of grid points in the XYZ of the grid box was set to 40 × 40 × 40 with a grid spacing of 0.375 Å. The number of genetic algorithm (GA) runs was set to 100, and the rest of the parameters retained their default values. Finally, the conformation with the lowest docking energy was further energy-minimized by the steepest descent method for 2000 steps first, followed by the conjugate gradient method for 2000 steps [30].

### 2.10. Images of Endogenous H_2_S in Live Cells

The hydrogen sulfide fluorescent probe SF7-AM was employed to detect the endogenous H_2_S in HA-VSMCs [11]. HA-VSMCs were cultured in DMEM supplemented with 10% (*v*/*v*) fetal bovine serum at 37 °C and 5% (*v*/*v*) CO_2_. When approximately 80% confluence was reached in a six-well plate, DTT or H_2_O_2_ was added to a final concentration of 400 μM. After an incubation for 30 min, the cells were washed three times with ice-cold PBS followed by the addition of fresh medium containing 2.5 μM SF7-AM. After an additional 30 min of incubation, the cells were washed with ice-cold PBS followed by the replacement of fresh medium and then imaged using an inverted fluorescence microscope (Leica, Germany). For VEGF stimulation, the cells were incubated with VEGF at a final concentration of 50 ng/mL for 30 min at 37 °C and then imaged. For the inhibitor experiments, the cells were preincubated with 2 mM PAG for 12 h to inhibit CSE activity. Subsequently, the cells were incubated with H_2_O_2_, DTT or VEGF for 30 min, washed and then imaged. Images were acquired for the cells in six different fields. Image analysis was performed using Image Pro Plus 6.0.

## 3. Results

### 3.1. Potential Disulfide Bond Redox Switch Regulates the Activity of CSE

**Figure 1:**
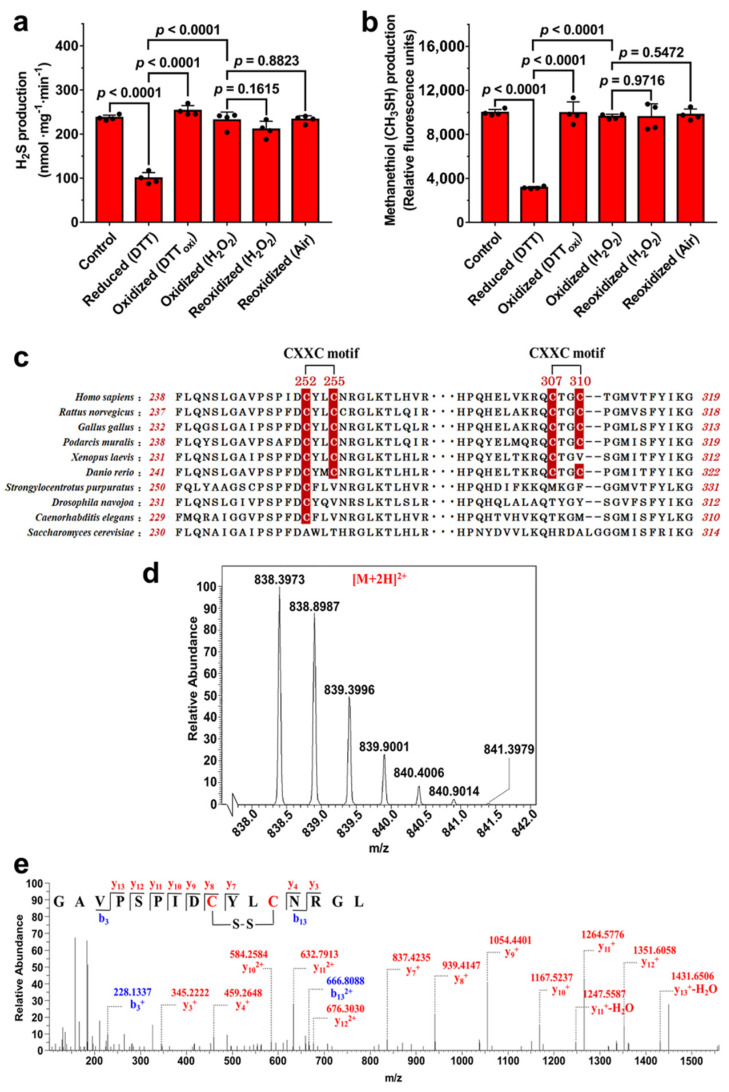
The redox state of CXXC motif in CSE affects its activity. (**a**,**b**) Recombinant wild-type CSE was incubated with 10 mM DTT, 5 mM oxidized DTT (DTT_oxi_), or 400 µM H_2_O_2_ for 1 h at room temperature. The samples were then ultrafiltered to remove DTT, DTT_oxi_ or H_2_O_2_. CSE activity was measured using 20 mM L-cysteine (**a**) or 10 mM *S*-methylcysteine (**b**) as substrates. The data points and errors are the means ± SD (*n* = 4). An unpaired, two-tailed Student’s *t* test was used to determine the significance of differences between two group means. Reduced (DTT): CSE treated with DTT; Oxidized (DTT_oxi_) and Oxidized (H_2_O_2_): CSE treated with DTT_oxi_ or H_2_O_2_; Reoxidized (H_2_O_2_) and Reoxidized (Air): reduced CSE was reoxidized by exposure to H_2_O_2_ or air oxygen. The specific activity of the reduced CSE using *S*-methylcysteine as a substrate (using DTNB detection) was 15.3 ± 1.22 nmol methanethiol mg^−1^ min^−1^. (**c**) Alignment of amino acid sequences of CSE from different species using the Unipro UGENE software. (**d**,**e**) LC-MS/MS analysis of the chymotrypsin-digested peptides containing the Cys252-Cys255 disulfide bond in CSE protein.

Previous studies have demonstrated that H_2_S production derived from CSE was increased by exposure of HUVECs to H_2_O_2_ or VEGF, implying that the activity of CSE is redox-regulated at the posttranslational level [10,11]. Based on the above analysis, the H_2_S-producing activities of the reduced CSE and the oxidized CSE were determined using L-cysteine as a substrate. The results showed the activity of the oxidized CSE was approximately 2.5 fold higher than the activity of the reduced CSE. When the reduced CSE was reoxidized by H_2_O_2_ or air oxygen, the activity of the reoxidized CSE was restored to a level similar to the level of the oxidized CSE (Figure 1a). In addition, to exclude the possible inference that substrate L-cysteine could reduce the oxidized CSE protein, *S*-methylcysteine was employed as the substrate to determine the methanethiol (CH_3_SH)-producing activity of CSE [12]. The reaction products of CH_3_SH can be measured using the fluorescent thiol probe CPM by fluorescence spectroscopy. The oxidized CSE had an approximately 3 fold increase in activity compared to the reduced CSE. After treatment of the reduced CSE with H_2_O_2_ or air oxygen, the activity of the reoxidized CSE was restored to a level similar to that of the oxidized CSE (Figure 1b).

Unlike human CSE protein, yeast CSE protein does not contain cysteine residue. To test whether yeast CSE can be redox regulated, the recombinant yeast CSE was expressed and purified. The specific activity of the purified CSE was 30.0 ± 2.15 nmol mg^−1^ min^−1^, and were approximately 8 fold lower than that of recombinant human CSE (236.8 ± 5.93 nmol mg^−1^ min^−1^). We next investigated the effect of various redox reagents on CSE activity. Expectedly, the H_2_S-producing activity of recombinant yeast CSE was not affected by treatment with DTT, DTT_oxi_, or H_2_O_2_ (Figure S2). These results suggested that the activity of human CSE can be reversibly regulated by redox modifications of reactive cysteines.

Human CSE contains 10 cysteine residues, four of which form two CXXC motifs, C252YLC255 and C307TGC310. Interestingly, these two CXXC motifs exist in vertebrates but not in microorganisms and invertebrates (Figure 1c), obviously related to the process of biological evolution. Through analysis of the tertiary structure of the reduced CSE, we speculated that the vicinal cysteine residues of the CXXC motifs could form a disulfide bond in the oxidized CSE. The number of thiols in CSE protein was determined using DTNB assay. Expectedly, a total of 9.9 ± 0.36 cysteines was modified per reduced CSE monomer, and the number decreased to 8.0 ± 0.24 in the oxidized CSE protein. These results indicated that the oxidized CSE protein contains a single disulfide bond.

### 3.2. Disulfide Bond Formation between Cys252 and Cys255 in CXXC Motif

To determine which cysteine residues form the disulfide bond in the CSE protein, the purified wild type CSE protein was analyzed using liquid chromatography tandem mass spectrometry (LC-MS/MS). In the resulting spectrum, a prominent 2^+^ charged peak (*m/z* 838.398) corresponding to the Cys252-Cys255 disulfide-containing chymotrypsin-digested peptide (observed molecular mass, 1674.781 Da; theoretical molecular mass, 1674.786 Da) was identified (Figure 1d,e). A trypsin-digested peptide containing the Cys252-Cys255 disulfide bond (observed molecular mass, 2207.042 Da; theoretical molecular mass, 2207.050 Da) was also observed (Figure S3). Additionally, the peptide containing the Cys307-Cys310 disulfide bond (observed molecular mass, 1527.693 Da; theoretical molecular mass, 1527.700 Da) was also identified (Figure S4). However, the relative ion abundance and the number of identified peptides containing the Cys252-Cys255 disulfide bond in mass spectrometric analysis were significantly higher than the relative ion abundance and the number of identified peptides containing a Cys307-Cys310 disulfide bond.

Additionally, the number of free thiols in CSE mutants was determined using DTNB assay. A total of 9.0 ± 0.14 cysteines were modified per oxidized C255S mutant monomer, similar to reduced C255S mutant (8.9 ± 0.23 thiols/monomer). In the case of C307S mutant enzyme, the number of free thiols was 6.9 ± 0.13 per oxidized C307S mutant monomer and 9.0 ± 0.24 per reduced monomer. Collectively, these results strongly indicated a reversible redox change in the vicinal cysteines of C252XXC255 motif (not C307XXC310 motif) involving a dithiol-disulfide reaction regulates the activity of CSE.

### 3.3. Residue Cys255 Is Crucial for Oxidation Sensing

Since the Cys252-Cys255 was the primary disulfide bond detected in the oxidized CSE, we speculated that the redox state of the C252XXC255 motif would regulate CSE activity. The C252A, C255A, C252S, C255S, C252S/C255S, C307S, and C310S forms of the enzyme were expressed in *E. coli*. However, no obvious expression of C252A, C255A and C252S/C255S mutants was detected. The H_2_S-producing activities of C252S and C255S mutant enzymes were 154.7 ± 6.42 and 137.1 ± 2.73 nmol mg^−1^ min^−1^, respectively, and were 1.4 and 1.6 fold lower than the wild type CSE (216.8 ± 10.15 nmol mg^−1^ min^−1^) (Figure S5). These results suggested that the cysteine residues in the CXXC motif are crucial for maintaining the activity of CSE.

**Figure 2:**
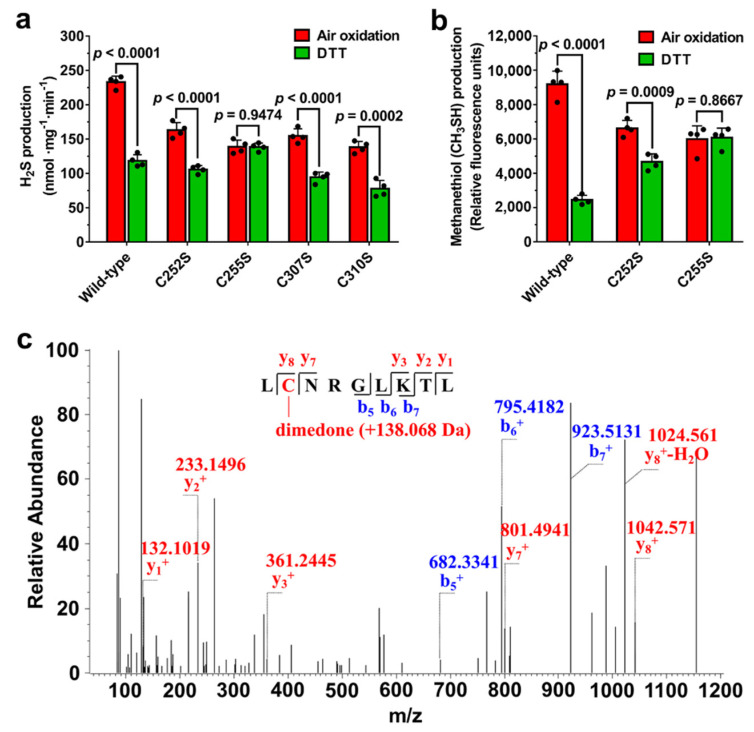
Sulfenic acid modification of the residue Cys255 is crucial for oxidation sensing. (**a**,**b**) Recombinant wild-type or mutant CSE was treated with 10 mM DTT. The samples were then ultrafiltered to remove DTT and exposed to air oxygen for 1 h at room temperature by gently pipetting up and down. CSE activity was measured using 20 mM L-cysteine (**a**) or 10 mM *S*-methylcysteine (**b**) as substrates. The data points and errors are the means ± SD (*n* = 4). An unpaired, two-tailed Student’s *t* test was used to determine the significance of differences between two group means. (**c**) LC-MS/MS analysis indicates the formation of the dimedone adduct (+138.068 Da) of sulfenic acid in Cys255.

We next determined the effect of these mutations in the C252XXC255 and C307XXC310 motifs on the activity of oxidized and reduced forms of the CSE protein. The activities of CSE were measured using l-cysteine or *S*-methylcysteine, respectively, as a substrate. The results showed that the activities of C252S, C307S and C310S mutant enzymes were redox regulated, but the activity ratio of the oxidized C252S mutant and the reduced C252S mutant was about 1.5, which is much lower than the activity ratio of the oxidized wild type CSE and the reduced wild type CSE. Unlike the C252S mutant enzyme, no change in the activity of the C255S mutant enzyme was observed after treatment with DTT or air oxidation (Figure 2a,b), indicating that Cys255 could be the primary amino acid residue which is involved in the response to oxidation. Disulfide bond can be formed via two general pathways: (a) thiol-disulfide exchange and (b) cysteine sulfenic acid as an intermediate. In the present study, the reduced CSE can be oxidized spontaneously in air-exposed aqueous solutions (Figure 2a,b), implying that the Cys252-Cys255 disulfide bond could be formed by the condensation of a thiol with sulfenic acid intermediate.

Previous studies have indicated that a cysteine-sulfenic acid (Cys-SOH) derivative can be formed spontaneously in air-exposed aqueous solutions of proteins in the absence of added oxidizing reagents [31,32,33,34]. In the present study, the reduced CSE protein solutions were exposed to air oxygen by gently pipetting up and down. Subsequently, cysteine sulfenic acids were specifically labelled with dimedone followed by mass spectrometric analysis. Mass spectrometric analysis showed the formation of the dimedone adduct (+138.068 Da) of sulfenic acid in Cys255, demonstrating the sulfenation of Cys255 in the C252S mutant protein (Figure 2c). However, sulfenic acid modification of Cys255 was not detected in the wild type CSE, suggesting that Cys255 could be oxidized to a sulfenic acid intermediate first, followed by reaction with the vicinal thiol group of Cys252 to form a stable Cys252-Cys255 disulfide bond. Overall, these results strongly indicated Cys255 is the primary redox-active site that initiates the disulfide bond formation in the response of oxidation.

### 3.4. Molecular Dynamics (MD) Simulations of the Reduced and Oxidized CSE

**Figure 3:**
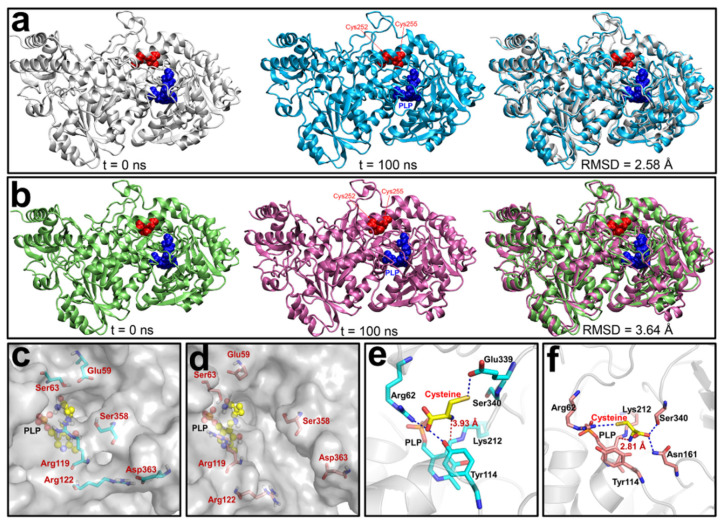
Structural comparison of the reduced and oxidized CSE during molecular dynamics (MD) simulations and molecular docking. (**a**) Snapshot structure of the reduced CSE at *t* = 0 ns (Left) and *t* = 100 ns (Center), along with the optimal backbone alignment of the two structures (Right). (**b**) Snapshot structure of the oxidized CSE at *t* = 0 ns (Left) and *t* = 200 ns (Center) along with the optimal backbone alignment of the two structures (Right). For clarity, the residues Cys252 and Cys255 in the CXXC motif are colored in red, and the PLP cofactor is colored in blue. (**c**,**d**) Comparison of the substrate entrance tunnel of the reduced CSE and the oxidized CSE after MD simulations. (**e**,**f**) Interaction between the substrate l-cysteine and the active center of the reduced and oxidized CSE using molecular docking. The blue dashed line denotes the hydrogen bond, and the red dashed line denotes the distance between the amino group of l-cysteine and the cofactor PLP.

To further investigate the molecular mechanisms underlying the redox regulation of CSE activity, crystallization of the oxidized CSE was attempted. However, all efforts were unsuccessful despite testing more than 2000 different conditions. Alternatively, to unveil how the Cys252-Cys255 disulfide bond could regulate the activity of CSE, we constructed an initial structural model of the oxidized CSE based on the crystal structure of the reduced CSE (PDB ID: 2NMP [9]. To investigate its relative stability and obtain a stable conformation of oxidized CSE, 100-ns MD simulations were carried out on oxidized and reduced forms of CSE. The time evolutions of the backbone root-mean-square deviation (RMSD) values of CSE from their initial positions (*t* = 0) were monitored (Figure S6). As shown in Figure 3, very little conformational change was observed in the reduced CSE after MD simulation, and the steady RMSD value in the reduced CSE was 2.58 Å (Figure 3a), suggesting that the conformation of the reduced CSE is relatively stable. However, the oxidized CSE displayed a large conformational change during MD simulation, and the corresponding RMSD value was 3.64 Å (Figure 3b), indicating that formation of the disulfide bond between Cys252 and Cys255 causes a significant structural disturbance to CSE.

A close view of structures indicated that the channel allowing the access of the substrate to the active site becomes larger in the oxidized form than in the reduced form owing to the increased distance of three pairs of amino acid residues (Glu59 and Ser63, Arg119 and Ser358, Arg122 and Asp363) located in the substrate entrance tunnel (Figure 3c,d and Figure S7). We next further investigated the interaction between the substrate l-cysteine and the active center of oxidized and reduced CSE using molecular docking. Expectedly, the free energy of l-cysteine binding to the active site of the oxidized CSE is −4.65 kcal/mol, lower than that of the reduced CSE (−4.02 kcal/mol), suggesting that the oxidized CSE has a stronger affinity to the substrate than the reduced CSE. Indeed, the orientation of the Ser340 side chain is reversed in the active center of oxidized CSE owing to the formation of the sulfide bond. Consequently, the substrate l-cysteine forms the hydrogen bond with Ser340 and Asn161 in the oxidized CSE, whereas in the reduced CSE the l-cysteine forms a stable hydrogen bond with Arg62 and Tyr114. The distance between the amino group of l-cysteine and the cofactor PLP is 3.93 Å in the reduced CSE, and the distance is greatly shortened to 2.81 Å in the oxidized CSE (Figure 3e,f). To investigate the effect of residue Ser340 on the redox regulation of CSE, the S340A mutant was expressed in *E. coli*. However, no soluble recombinant protein was detected, implying that Ser340 is crucial for maintaining an active enzyme conformation. Collectively, these results explain why the activity of the oxidized form of CSE is higher than the activity of the reduced form.

### 3.5. Oxidative Stress Stimulates H_2_S Production via Enhancement of CSE Activity in HA-VSMCs

**Figure 4:**
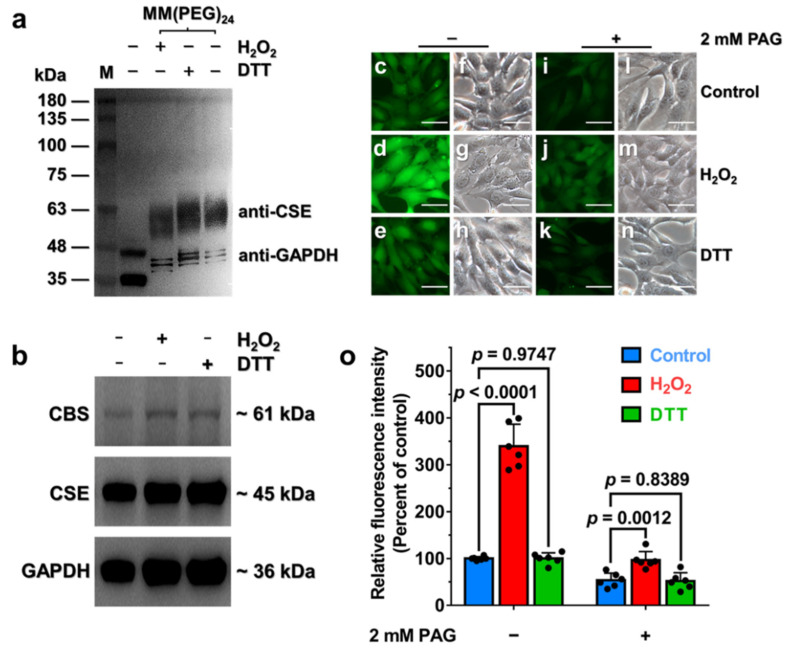
Increasing activity of CSE in its oxidized state enhances the H_2_S signaling in live HA-VSMCs. (**a**) Cells were exposed to 0.4 mM H_2_O_2_ or 0.4 mM DTT for 30 min, followed by washing three times with ice-cold PBS buffer and were treated with 10% (*v*/*v*) trichloroacetic acid immediately at 4 °C for 30 min before being alkylated with 20 mM MM(PEG)_24_. The samples were resolved on SDS-PAGE and immunoblotted for CSE. (**b**) Cells were exposed to 0.4 mM DTT or 0.4 mM H_2_O_2_ for 30 min. The cell lysates were resolved on SDS-PAGE and immunoblotted for CBS and CSE. (**c**) Untreated cells imaged as described in methods and were used as a control group. (**d**), Cells were incubated with 0.4 mM H_2_O_2_ for 30 min at 37 °C and then imaged. (**e**) Cells were incubated with 0.4 mM DTT for 30 min at 37 °C and then imaged. (**f**–**h**) Brightfield images corresponding to (**c**–**e**). (Scale bar, 250 μm). (**i**–**k**) Cells were preincubated with 2 mM PAG for 12 h to inhibit CSE activity. Subsequently, the cells were incubated with PBS (**i**), 0.4 mM H_2_O_2_ (**j**) or 0.4 mM DTT (**k**) for 30 min, washed and then imaged. (**l**–**n**) Brightfield images corresponding to (**i**–**k**) (Scale bar, 250 μm). (**o**) Quantification of the fluorescence microscope images of H_2_S signaling in HA-VSMCs, with data from (**c**–**e**,**i**–**k**) for comparison. The graph represents the relative fluorescence intensity compared with the relative fluorescence intensity of untreated cells (**c**) and shows the means ± SD (*n* = 6). An unpaired two-tailed Student’s *t* test was used to determine the significance of differences between the two group means.

To determine whether the C252XXC255 motif in CSE exists as the reduced or oxidized form, the free thiol groups of proteins in HA-VSMCs lysates were labeled with MM[PEG]_24_, which has a molecular mass of 1240 Da. The cells were incubated with 400 μM H_2_O_2_ or 400 μM DTT to induce oxidative or reductive stress, respectively (Figure S8). As shown in Figure 4a, the average molecular masses of labeled CSE under oxidative stress were slightly less than the average molecular masses of labeled CSE under reduced stress, suggesting that the number of free thiols in CSE is decreased in cell lysates from oxidatively challenged cells versus DTT-treated cells, and the disulfide bond in CSE should be formed. Additionally, glyceraldehyde-3-phosphate dehydrogenase (GAPDH), which was used as a loading control, also exists in reduced and oxidized states in HA-VSMCs (Figure 4a). Notably, however, the PEGylated samples ran as a smeared and broadened band which is probably due to the heterogeneity of labeled protein and the interaction between PEG and SDS [17,35,36]. These results demonstrated that the C252XXC255 motif in CSE exists in reduced and oxidized states and this motif can be influenced by relevant cellular redox stresses.

As the activity of recombinant CSE was regulated via the redox-active disulfide bond formed in the C252XXC255 motif, we anticipated that formation of the disulfide bond in C252XXC255 motif would stimulate the activity of CSE to concomitantly enhance CSE-derived H_2_S production. Compared with a very low level of expression of CBS, CSE is highly expressed in HA-VSMCs as evidenced by western blot analysis (Figure S9), indicating that CSE is the major enzyme that catalyzes the biosynthesis of H_2_S in HA-VSMCs. These results are consistent with previous reports [37,38]. Additionally, the mRNA and protein levels of CSE were not significantly changed when cells were incubated with 400 μM H_2_O_2_ or 400 μM DTT for 30 min (Figure 4b and Figure S10).

We next investigated the change of endogenous H_2_S production, which was detected using a fluorescent probe SF7-AM, in live HA-VSMCs that were exposed to exogenous H_2_O_2_ or DTT. As shown in Figure 4c–h, the H_2_O_2_-treated cells displayed a great increase in intracellular fluorescence compared with the control, whereas the fluorescent intensity was not significantly affected when the cells were incubated with DTT. To validate the contribution of CSE to the stimulation of the H_2_S production, the cells were incubated with DL-propargylglycine (PAG), a specific inhibitor of CSE. The results indicated that the increase in the fluorescent intensity was remarkably decreased when the cells were exposed to H_2_O_2_ (Figure 4i–o). Collectively, these results demonstrated that H_2_O_2_-induced oxidative stress caused the increase in the specific activity of CSE in mammalian cells through thiol oxidation with the concomitant enhancement of the intracellular H_2_S production.

### 3.6. Endogenous H_2_O_2_ Triggered by VEGF Promotes Cellular H_2_S Production via the Enhancement of CSE Activity

**Figure 5:**
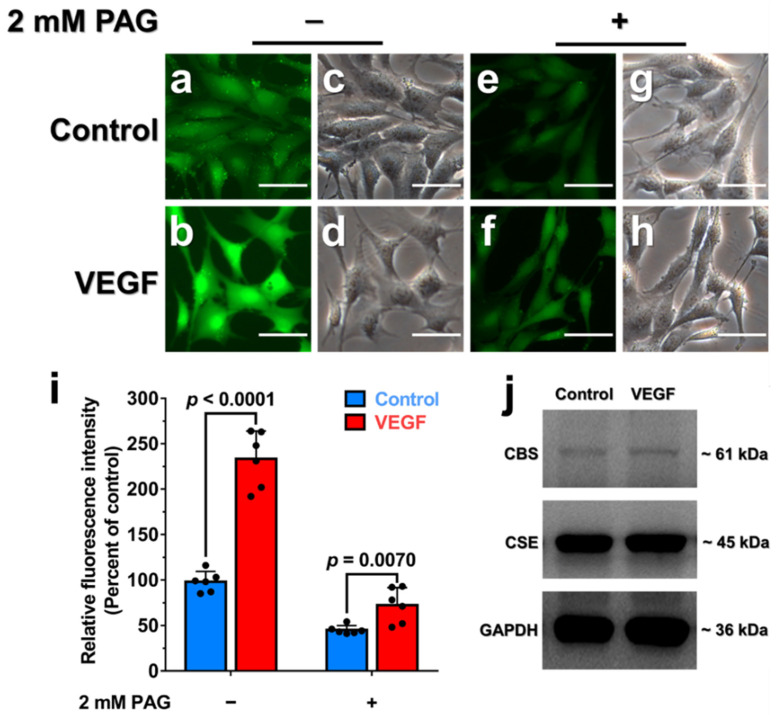
The fluorescence microscope images of H_2_S signaling in HA-VSMCs treated with VEGF. (**a**) Untreated cells imaged as described in methods and were used as a control group. (**b**) Cells were incubated with VEGF at a final concentration of 50 ng/mL for 30 min at 37 °C and then imaged. (**c**,**d**) Brightfield images corresponding to (**a**,**b**) (Scale bar, 250 μm). (**e**,**f)** Cells were preincubated with 2 mM PAG for 12 h to inhibit CSE activity. Subsequently, the cells were incubated with PBS (**e**) or 50 ng/mL VEGF (**f**) for 30 min, washed and then imaged. (**g**,**h**) Brightfield images corresponding to (**e**,**f**) (Scale bar, 250 μm). (**i**) Quantification of the fluorescence microscope images of H_2_S signaling in HA-VSMCs, with data from (**a**,**b**,**e**) and (**f**) for comparison. The graph represents the relative fluorescence intensity compared with the relative fluorescence intensity of the untreated cells (**a**) and shows the means ± SD (*n* = 6). (**j**) Cells were exposed to 50 ng/mL VEGF for 30 min at 37 °C. The cell lysates were resolved on SDS-PAGE and immunoblotted for CBS and CSE. Untreated cells were used as a control group.

We next investigated the effect of endogenous H_2_O_2_ on CSE-derived H_2_S production in HA-VSMCs. Previous studies indicated that vascular endothelial growth factor (VEGF) stimulation of VEGFR2 can trigger an increased level of NADPH oxidases (NOX)-derived H_2_O_2_ and the concomitant enhancement of H_2_S production [11,39,40,41], but the mechanism involving the crosstalk between H_2_O_2_ and H_2_S remains incompletely understood. Indeed, the increased level of cellular H_2_O_2_ production was determined in the presence of VEGF or exogenous H_2_O_2_ (Figure S11). As expected, the H_2_S production was also dramatically increased in HA-VSMCs that were incubated with 50 ng/mL VEGF for 30 min (Figures S12 and S13), but the addition of 2 mM PAG dramatically inhibited the production of endogenous H_2_S in cells (Figure 5a–i). Moreover, incubation with VEGF did not significantly enhance the H_2_S production in the presence of PEG-catalase, an enzymatic cell-permeable H_2_O_2_ scavenger with high H_2_O_2_ specificity (Figure S14). As an additional control, incubation with 400 μM exogenous H_2_O_2_ displayed no significant increase in H_2_S production in HA-VSMCs pretreated with PEG-catalase (Figure S14). Additionally, the mRNA and protein levels of CSE and CBS were not significantly changed when cells were incubated with 50 ng/mL VEGF for 30 min (Figure 5j and Figure S15). Taken together, these experiments indicated that endogenous H_2_O_2_ triggered by VEGF can stimulate CSE activity via posttranslational modification of CSE with the concomitant enhancement of the intracellular H_2_S production.

## 4. Discussion

CBS and CSE are two critical enzymes involved in the synthesis of cysteine and glutathione in the trans-sulphuration pathway and play a key role for maintaining the cellular redox homeostasis [42,43,44]. Alternatively, CBS and CSE are the main H_2_S-generating enzymes in a tissue-specific manner [45]. However, compared with CBS, the redox regulation of CSE remains poorly understood. Since western blot analysis had revealed that CSE protein levels were unchanged in cell lysates from oxidatively challenged cells versus untreated cells, a posttranslational mechanism for upregulation of the CSE/H_2_S system was implicated [11]. In this study, we report that cellular oxidative stress stimulates CSE-derived H_2_S synthesis through the oxidation of free thiols in the C252XXC255 motif to form the disulfide bond.

CSE contains two highly conserved CXXC motifs, which exist in reduced states in the crystal structure (PDB: 2NMP). The structure of human CSE reveals that C307XXC310 is located on the periphery of the enzyme, while C252XXC255 is located near the dimer interface and is not particularly well exposed [9]. CXXC motifs are common in the catalytic centers of the enzymes performing disulfide isomerization or redox reactions. More recently, Scott Smith reported that the C307XXC310 motif in CSE may act as a secondary active site responsible for the reduction of homocysteine [46], but the catalytic mechanisms and the physiological relevance are unclear. Our previous study indicated that the activity of CBS is posttranslationally regulated by a redox-active disulfide bond in the CXXC motif [17]. In this study, we found that CSE activity is regulated by a disulfide redox switch in the C252XXC255 motif. The C307XXC310 motif, another CXXC motif in CSE, cannot be readily oxidized to form a disulfide bond, and its functions, if it has functions, need to be further investigated.

The reduced C252S mutant can be oxidized by exposure it to air oxygen, and the activity of the C252S mutant in the oxidized state was 1.5 fold higher than its activity in the reduced state. However, treatment of the C255S mutant with various redox reagents did not significantly affect its activity (Figure 2a,b). These results indicated that the residue Cys255 in C252XXC255 motif plays a key role in oxidation sensing, and the free thiol of Cys255 could be oxidized to stable cysteine-sulfenic acid (Cys-SOH) by exposing it to air oxygen. Expectedly, sulfenic acid modification of Cys255, which was detected by mass spectrometric analysis (Figure 2c), could directly cause the increased activity of the C252S mutant to some extent. Sulfenic acids exhibits both electrophilic and nucleophilic reactivity and are usually unstable and highly reactive [47]. However, stable sulfenic acid modifications have been identified in some redox-regulated proteins [32,47,48]. The stabilization of protein sulfenic acids requires the absence of vicinal protein thiols to prevent disulfide bond formation [49]. Indeed, the stable sulfenic acid modification of Cys255 was detected in the C252S mutant, but not in the wild type CSE, implying that the sulfenic acid formed by oxidizing the thiol group of Cys255, which immediately reacts with the vicinal Cys252 to form a stable disulfide bond.

Although the C252XXC255 motif is located near the dimer interface and relatively buried in the interior of the CSE protein, protein dynamics could allow access to this reactive cysteine residue. Accordingly, small molecule oxidants such as H_2_O_2_ under physiological conditions should readily oxidize the thiol group of Cys255. Changes in the redox state of the C252XXC255 motif lead to the conformational changes in the active site of CSE and the concomitant influence on CSE activity by employing MD simulation and molecular docking (Figure 3). Indeed, mutagenesis of cysteine residues in the C252XXC255 motif remarkably affects the specific activity of CSE (Figure S5). In untreated cells, the redox-active thiols in CSE exists predominantly in the reduced state (Figure 4a). When the cells are exposed to exogenous H_2_O_2_, the oxidation of the redox-active thiols in the C252XXC255 motif stimulates the activity of CSE and the concomitant enhancement of the intracellular H_2_S production. It is not known whether or not the disulfide bond can be reduced enzymatically or non-enzymatically by antioxidants, and this issue needs to be investigated.

As with the CSE protein, human CBS, which is another key enzyme involved in the biosynthesis of H_2_S, also contains a C272XX275C redox active-site motif. Our previous study demonstrated that reduced CBS has higher H_2_S-producing activity in HEK293 cells exposed to reductive challenge [17]. At first glance, these results seem contradictory to the observation that the oxidation of the C252XXC255 motif enhances the activity of human CSE and the intracellular H_2_S production in HUVECs. However, the expression of CSE and CBS is tissue-specific; CSE is mostly expressed in the cardiovascular system and other big tissues, such as the kidney and liver, and CBS is in brain [3,4,50]. As a result, the contribution of CBS and CSE to H_2_S production in different tissues and cells could be quite different. Indeed, CBS is the major enzyme that catalyzes the synthesis of H_2_S in HEK293 cells [17], but in HUVECs CSE is the principal enzyme responsible for the endogenous production of H_2_S. Therefore, the changes of endogenous H_2_S level may depend on the redox regulation of the predominant H_2_S-generating enzyme (CBS or CSE) in different tissues and cells under oxidative/reductive conditions.

**Figure 6:**
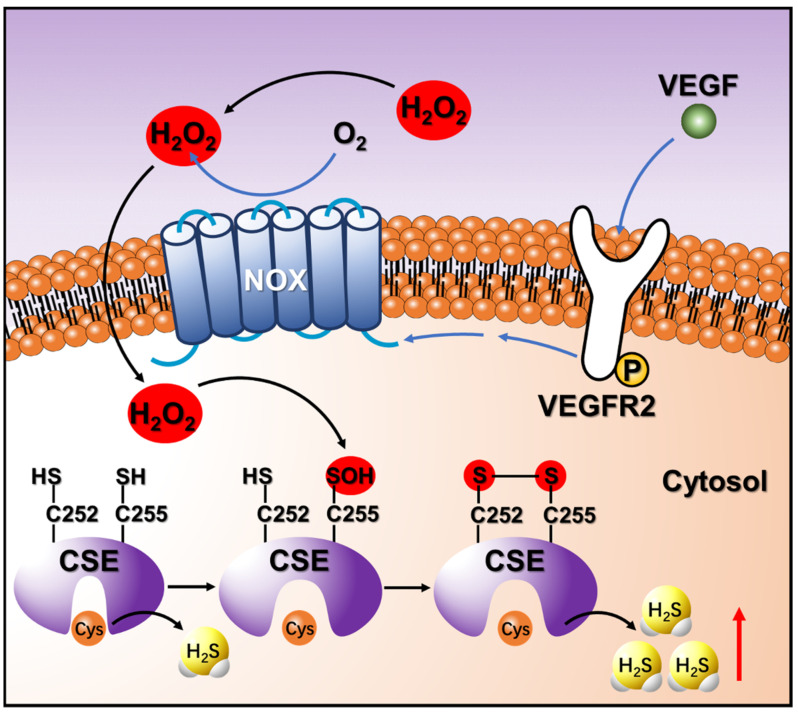
Schematic of the regulatory mechanism of CSE and H_2_O_2_/H_2_S crosstalk in HA-VSMCs stimulated with exogenous H_2_O_2_ or VEGF. VEGF stimulates the VEGFR2 receptor, which is autophosphorylated and activates NOX to generate endogenous H_2_O_2_. Exogenous H_2_O_2_ or endogenous H_2_O_2_ triggered by VEGF oxidizes the thiol group of Cys255 to the sulfenic acid, which stimulates the activity of CSE, thus leading to the enhancement of H_2_S production. The unstable sulfenic acid of Cys255 reacts quickly with the thiol of the vicinal Cys252 to form the stable disulfide bond.

Vascular endothelial growth factor (VEGF) is an angiogenic factor that regulates angiogenesis by inducing proliferation, migration, and permeability of endothelial cells [51,52]. Previous studies indicated stimulation of HUVECs with VEGF increased H_2_S production, and this process is dependent on NOX-derived H_2_O_2_ [11]. However, the mechanism involving the crosstalk between H_2_O_2_ and H_2_S remains unclear. In this report, we have provided clear evidence that changes in the redox state of the C252XXC255 motif regulate the activity of CSE, and Cys255 is the primary redox-active site that initiates the disulfide bond formation in the response to oxidative stress. Moreover, we have discovered that exogenous H_2_O_2_ and endogenous H_2_O_2_ triggered by VEGF can stimulate the activity of CSE in HA-VSMCs by sulfenylation of Cys255 leads to a sulfenic acid intermediate that spontaneously forms an intramolecular disulfide bond in the C252XXC255 motif, thus leading to the enhancement of H_2_S production (Figure 6). Increased endogenous H_2_S has been shown to protect against oxidative stress in HUVECs and in brain cells [53,54,55]. Additionally, protein *S*-sulfhydration is a newly discovered post-translational modification of specific cysteine residue(s) in target proteins by H_2_S. Enhancement of cellular H_2_S production under oxidative stress could induce *S*-sulfhydration on enzymes or receptors, which is involved in a broad range of cellular functions and metabolic pathways [56,57]. In a more general sense, this study provides a mechanism by which H_2_O_2_-mediated oxidative stress promotes the activity of CSE and increases the H_2_S production in the response to physiological oxidative stress.

## 5. Conclusions

In summary, we report a new posttranslational modification of CSE that provides a molecular mechanism for H_2_O_2_/H_2_S crosstalk. CSE activity is controlled by the redox state of the C252XXC255 motif, and the residue Cys255 is crucial for oxidation sensing in the response to H_2_O_2_-induced oxidative stress under physiological conditions.

## Data Availability

Data is contained within the article or supplementary files.

## Supplementary Materials

The following are available online at https://www.mdpi.com/article/10.3390/antiox10091488/s1.

## Abbreviations

CSE: cystathionine *γ*-lyase; CBS, cystathionine *β*-synthase; 3-MST, 3-mercaptopyruvate sulfur transferase; HA-VSMCs, human aortic vascular smooth muscle cells; HUVECs, human umbilical vein endothelial cells; VEGF, vascular endothelial growth factor; PLP, pyridoxal 5′-phosphate; DTT, dithiothreitol; PAG, DL-propargylglycine; LC-MS/MS, liquid chromatography tandem mass spectrometry; MM(PEG)_24_, methyl-PEG-maleimide; SDS-PAGE, sodium dodecyl sulfate–polyacrylamide gel electrophoresis; TCA, trichloroacetic acid; NADPH, nicotinamide adenine dinucleotide phosphate; CPM, 7-Diethylamino-3-(4-maleimidophenyl)-4-methylcoumarin; DTNB, 5,5′-dithiobis-2-nitrobenzoic acid; MD, molecular dynamics; RMSD, root-mean-square deviation
