# Supplementary material for: H2O2-Mediated Oxidative Stress Enhances Cystathionine γ-Lyase-Derived H2S Synthesis via a Sulfenic Acid Intermediate
Source: Antioxidants (Basel). 2021 Sep 18;10(9):1488. doi: 10.3390/antiox10091488 (PMC8466214; doi:10.3390/antiox10091488)
Supplement: Supplementary file 1 [file antioxidants-10-01488-s001.zip › antioxidants-1361965-supplementary.pdf]

Supplementary material

H_2O_2-mediated oxidative stress enhances cystathionine *γ*-lyase-derived H_2S synthesis *via* a sulfenic acid intermediate

Jun Wang#, Guanya Jia#, Heng Li, Shasha Yan, Jing Qian, Xin Guo, Ge Li, Haizhen Qi, Zhilong Zhu, Yanjun Wu, Weijuan He and Weining Niu*

School of Life Sciences, Northwestern Polytechnical University, Xi'an, 710072, China

* Correspondence: niuweining@nwpu.edu.cn.

# These authors contributed equally to this paper.

Keywords: cystathionine *γ*-lyase, hydrogen sulfide, hydrogen peroxide, sulfenic acid modification, oxidative stress

1. Methods

1.1 Expression and purification yeast cystathionine-γ-lyase

The gene fragment encoding yeast cystathionine-γ-lyase (CSE) was amplified from the genome of *Saccharomyces cerevisiae* and cloned into the pET28a plasmid to generate N-terminal histidine-tagged proteins. *E. coli* BL21(DE3) cells with the yeast CSE expression constructs were grown in LB medium containing kanamycin (50 μg/mL) until the optical density at 600 nm (OD_{600}) reached 0.6-0.8. The expression of yeast CSE was induced by adding 0.1 mM isopropyl β-D-1-thiogalactopyranoside (IPTG), and the cells were further cultured for 12 h at 30 °C.

The harvested cells were resuspended in lysis buffer containing 50 mM phosphate-buffered saline (PBS, pH 7.4), 300 mM NaCl, 100 mg/L lysozyme, and an "EDTA-free" protease inhibitor tablet (Roche). The resuspended cells were then incubated on ice for 1 h and sonicated with Ultrasonic Cell Disruption System (Scientz, Ningbo, China). The supernatant was collected by centrifugation at 12000 rpm for 30 min at 4 °C and loaded on a HisTrap FF column (GE Healthcare, USA) that had been equilibrated with 30 mM imidazole and 300 mM NaCl in 50 mM PBS, pH 7.4. The recombinant yeast CSE was eluted with 300 mM imidazole in 50 mM PBS, pH 7.4. The collected fractions were desalted using a HiPrep desalting column (GE Healthcare, USA) that had been equilibrated with 50 mM PBS, pH 7.4. The purified protein was stored at -80 °C and the purity was assayed by SDS-PAGE analysis.

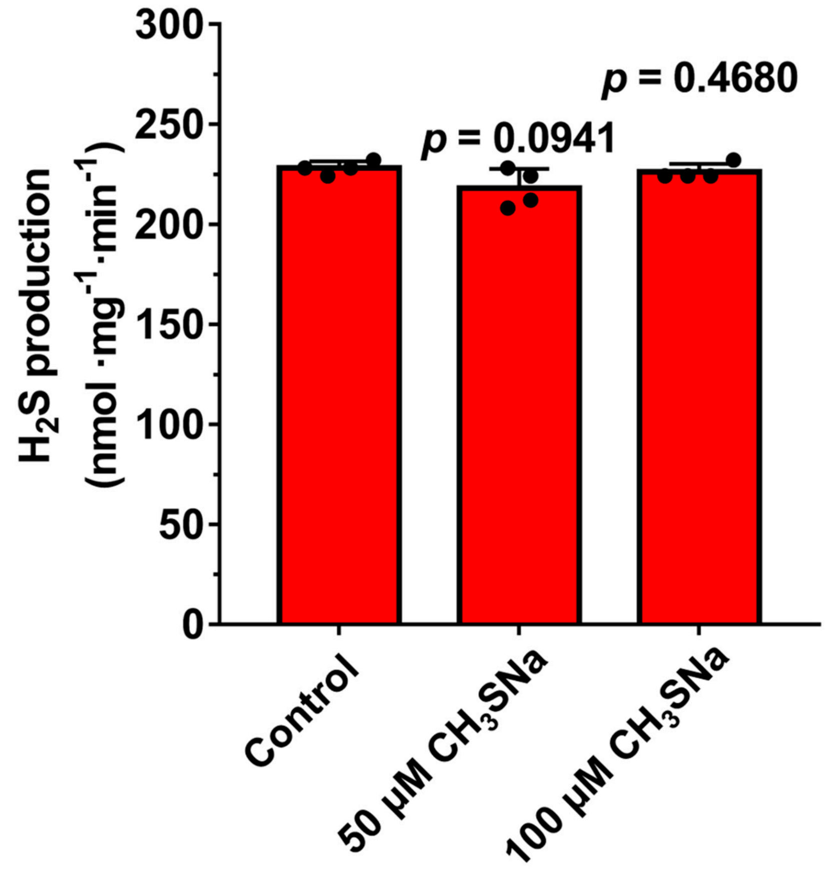

Figure S1. The effect of methanethiol (CH_3SH) on the activity of wild-type CSE. Recombinant wild-type CSE was incubated with different concentrations of sodium thiomethoxide (CH_3SNa) for 10 min at 37 °C, respectively. CSE activity was measured using 20 mM *L*-cysteine as described in **Materials and methods.** The data points and errors are the means ± SD (n=4). An unpaired, two-tailed Student's *t* test was used to determine the significance of differences between two group means. *p*-values *versus* Control group.

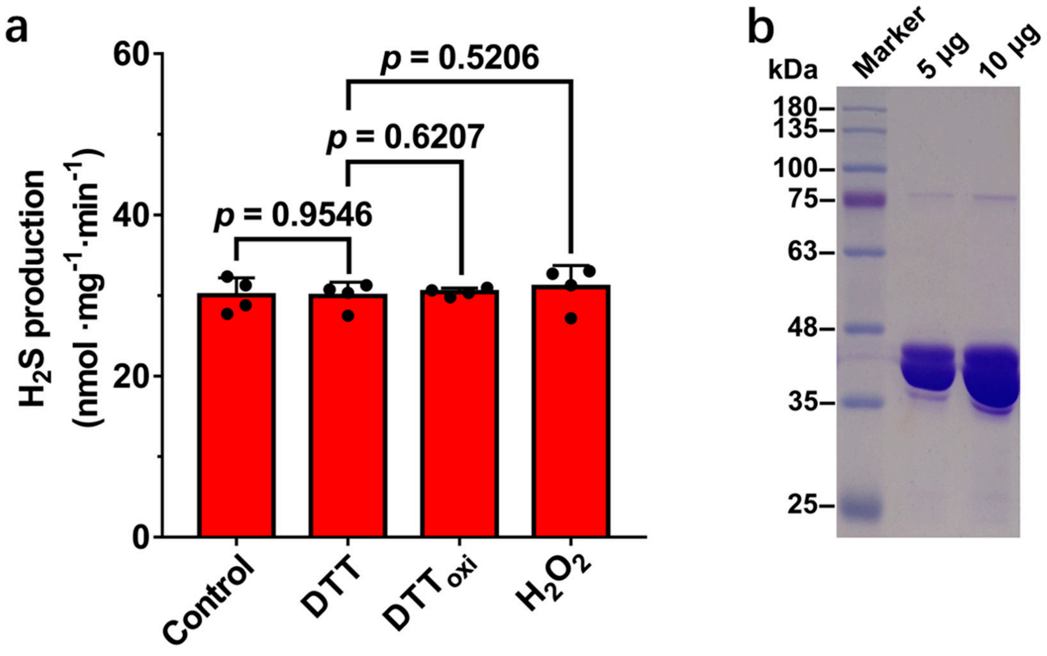

Figure S2. The effect of various redox agents on the activity of yeast cystathionine-*γ*-lyase (CSE). a, Recombinant yeast CSE was incubated with 10 mM DTT, 5 mM oxidized DTT (DTT_{oxi}) or 400 μM H_2O_2 for 1 h at room temperature. The samples were then ultrafiltered to remove DTT, DTT_{oxi} or H_2O_2. CSE activity was measured using 20 mM *L*-cysteine as substrates as described in **Materials and methods**. The data points and errors are the means ± SD (n=4). An unpaired, two-tailed Student's *t* test was used to determine the significance of differences between two group means. **b,** The purified recombinant protein was analyzed by SDS-PAGE stained with Coomassie Blue.

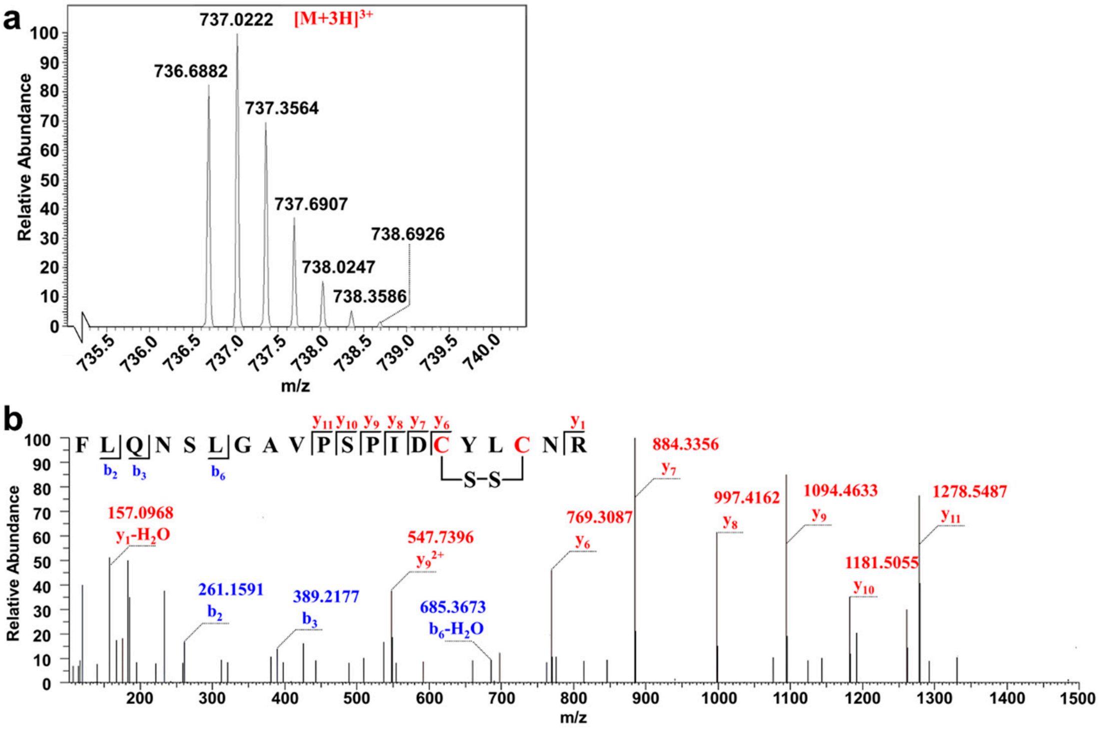

Figure S3. LC-MS/MS analysis of the trypsin digested peptides containing the Cys252-Cys255 disulfide bond in CSE protein. a, The recombinant CSE protein was analyzed by LC-MS/MS as described in **Materials and methods**. The monoisotopic mass $[M+3H]^{3+}$ of this peptide was 736.688 Da (observed nominal mass =2207.042 Da; expected nominal mass = 2207.050 Da). **b**, The mass spectrum of the FLQNSLGAVPSPID<u>C</u>YL<u>C</u>NR peptide that contained the oxidized form of the C252XXC255 motif in CSE.

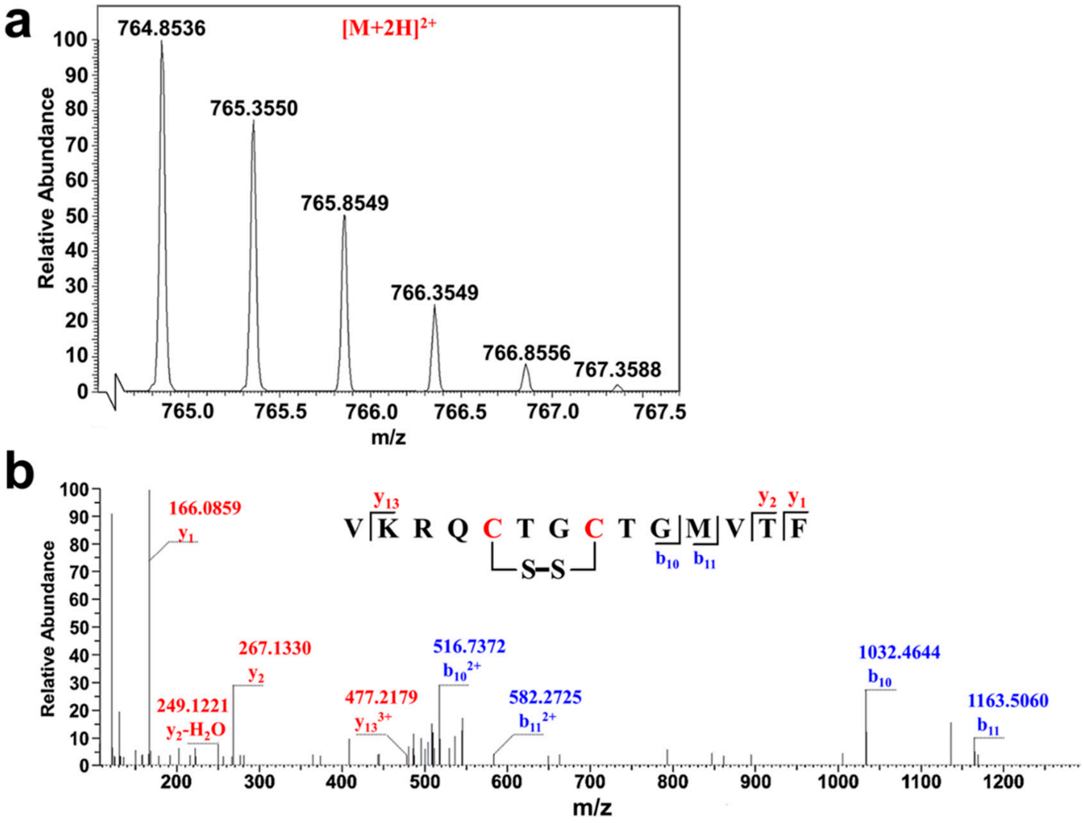

Figure S4. LC-MS/MS analysis of the chymotrypsin digested peptides containing the Cys307-Cys310 disulfide bond in CSE protein. a, The recombinant CSE protein was analyzed by LC-MS/MS as described in **Materials and methods**. The monoisotopic mass $[M+2H]^{2+}$ of this peptide was 764.854 Da (observed nominal mass =1527.693 Da; expected nominal mass = 1527.700 Da). **b**, The mass spectrum of the VKRQ<u>C</u>TG<u>C</u>TGMVTF peptide that contained the oxidized form of the C307XXC310 motif in CSE.

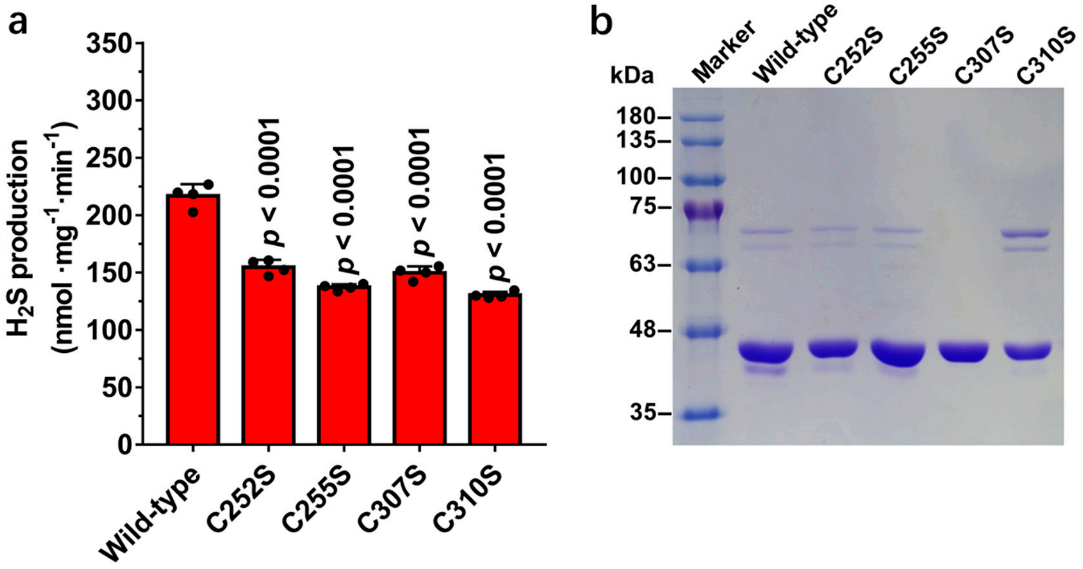

Figure S5. Activities of the recombinant human wild-type CSE and mutants. a, Activities of wild-type CSE, CSE(C252S), CSE(C255S), CSE(C307S) and CSE(C310S) were determined as described using 20 mM *L*-cysteine as substrates as described in **Materials and methods**. The data are presented as the means ± SD (n=4). An unpaired two-tailed Student's *t* test was used to determine the significance of differences between the two group means. $p < 0.0001$ *versus* wild-type CSE. **b,** The purified recombinant protein was analyzed by SDS-PAGE stained with Coomassie Blue.

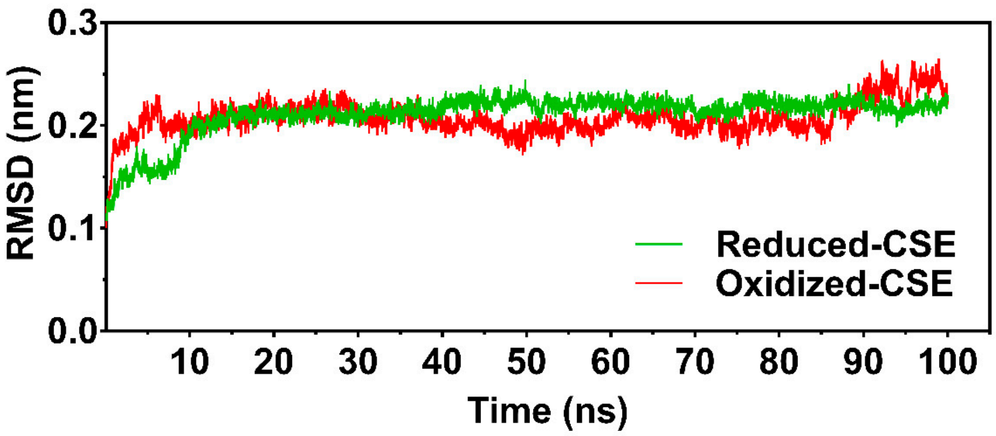

Figure S6. Changes in RMSD values of Reduced-CSE and Oxidized-CSE during molecular dynamics (MD) simulation. All MD simulations were carried out for 100 ns using the GROMACS 2018.4 package with constant temperature, pressure and periodic boundary conditions. The time evolutions of the backbone RMSD values of CSE from their initial positions (t = 0) were monitored.

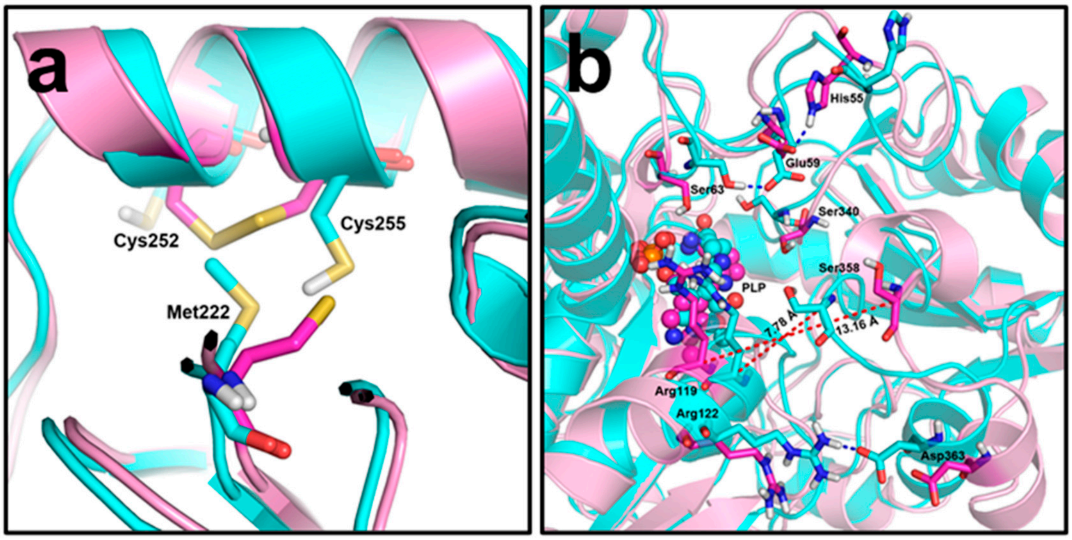

Figure S7. Structural comparison between oxidized-CSE (pink) and reduced-CSE (blue) after molecular dynamics simulation. a, Close view of Cys252 and Cys255 in the oxidized and reduced CSE; **b,** Comparison of the substrate entrance tunnel of the oxidized and reduced CSE after MD simulations. Blue dashed line denotes the hydrogen bond, red dashed line denotes the distance of Cα atoms between two amino acid residues, and the ball-and-stick model denotes the cofactor PLP.

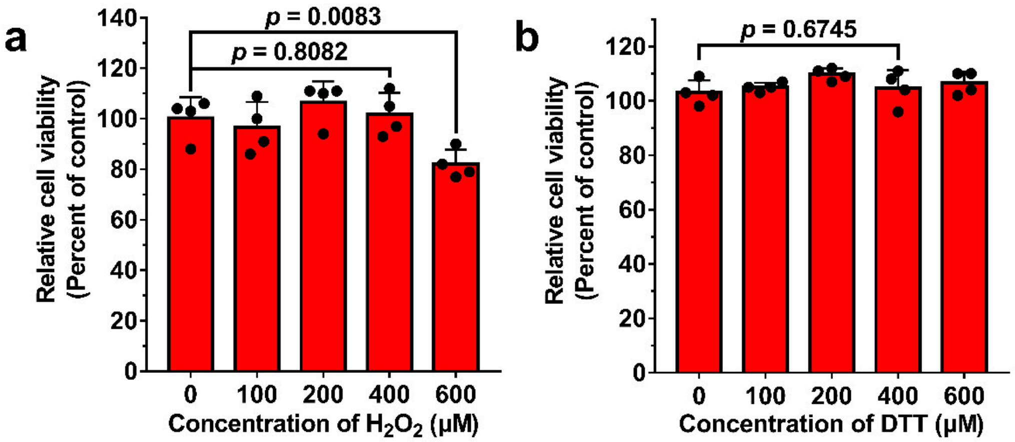

Figure S8. Effect of varied concentration of H_2O_2 and DTT on cell viability of HA-VSMCs. The effect of varied concentrations of H_2O_2 (**a**) and DTT (**b**) on cell viability was evaluated by CCK-8 assay (Dojindo, Shanghai, China) according to the instructions provided by the manufacturer. The data are presented as the means ± SD (n=4). An unpaired two-tailed Student's *t* test was used to determine the significance of differences between two group means.

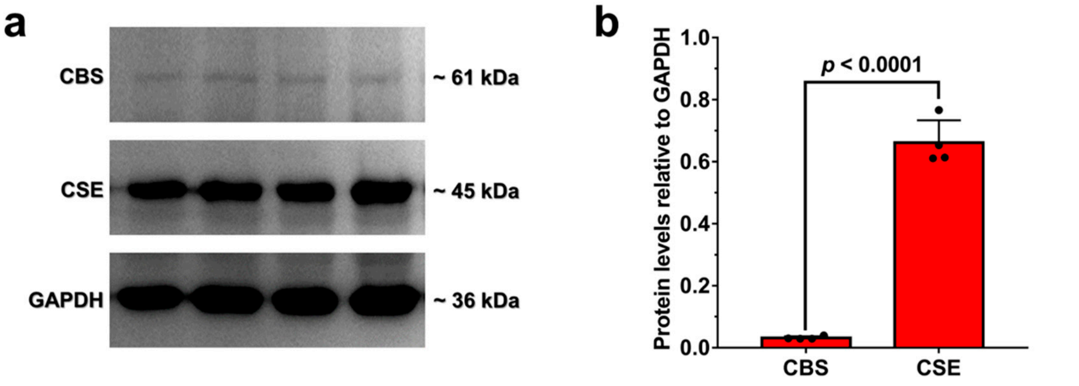

Figure S9. CSE is the principal enzyme responsible for the endogenous production of H_2S in HA-VSMCs. HA-VSMCs were cultured to 80% confluence, and then cell lysates were resolved on SDS-PAGE and immunoblotted for CBS, CSE. Glyceraldehyde-3-phosphate dehydrogenase (GAPDH) was used as a loading control.

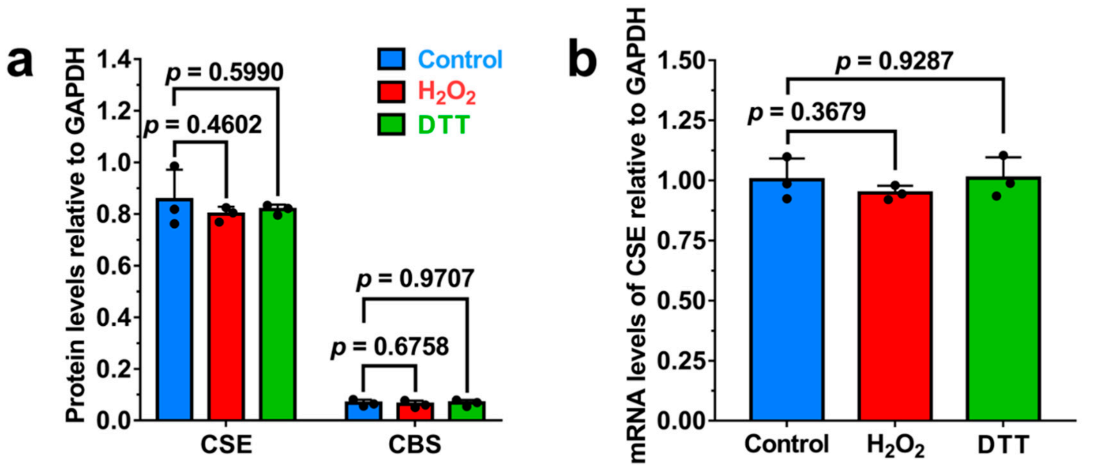

Figure S10. The effect of H_2O_2 and DTT on the mRNA and protein levels of CSE in HA-VSMCs. a, HA-VSMCs were cultured to 80% confluence, and then were incubated with 400 µM H_2O_2 or 400 µM DTT for 30 min. Cell lysates were resolved on SDS-PAGE and immunoblotted for CBS, CSE. Glyceraldehyde-3-phosphate dehydrogenase (GAPDH) was used as a loading control. **b,** Cells were incubated with 400 µM H_2O_2 or 400 µM DTT for 30 min, and then the cells were harvested for RNA extraction and cDNA reverse transcription followed by qPCR analysis using SYBR Mix from Agbio (Changsha, China). The level of GAPDH was used as an internal reference gene. The data are presented as the means ± SD (n=3). An unpaired two-tailed Student's *t* test was used to determine the significance of differences between two group means.

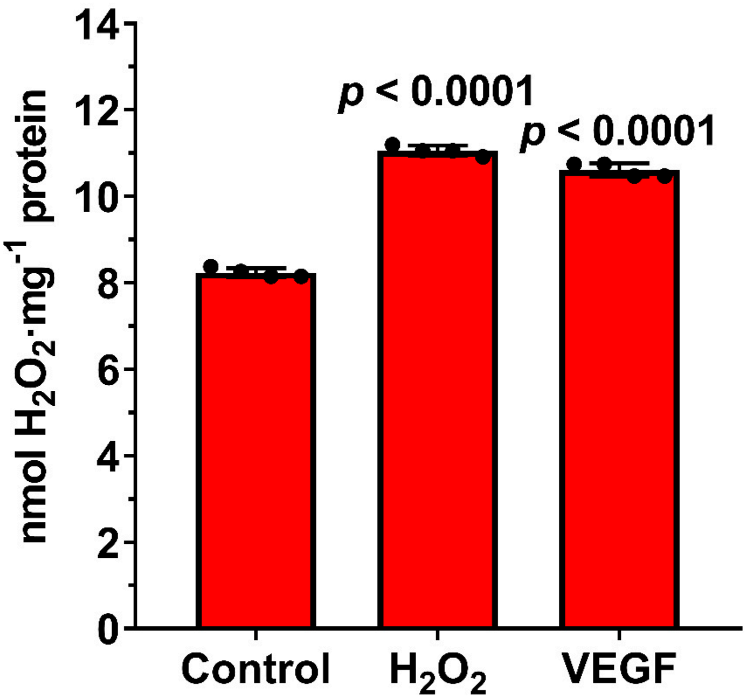

Figure S11. Determination of cellular H_2O_2 production in HA-VSMCs. Cells were incubated for 10 min with 400 μM H_2O_2 or 50 ng/ml VEGF, and then the cells were harvested for determining H_2O_2 production using a Hydrogen Peroxide Assay Kit (Beyotime, Shanghai, China). The columns represent the means ± SD (n=4). An unpaired two-tailed Student's *t* test was used to determine the significance of differences between two group means. $p < 0.0001$ *versus* Control group.

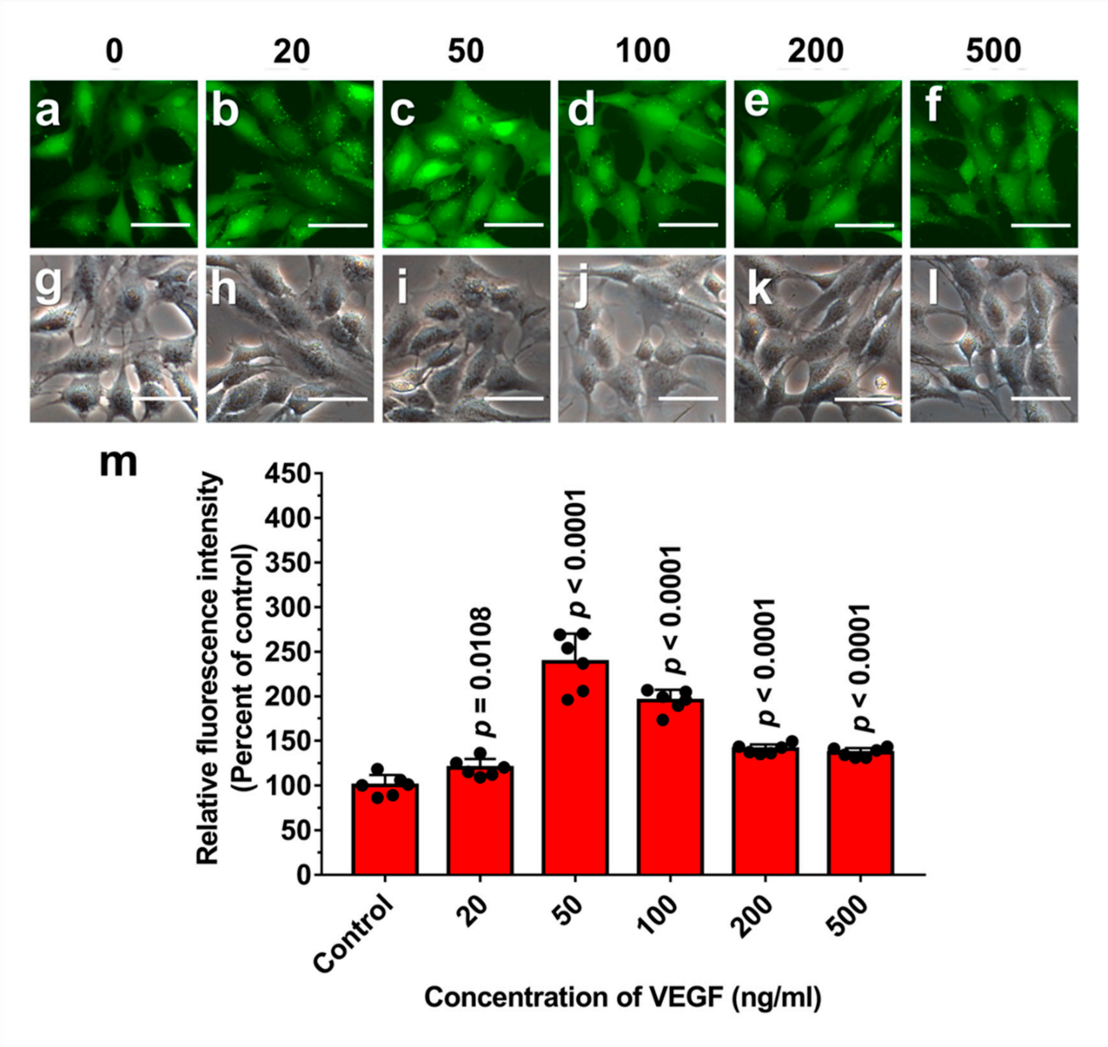

Figure S12. Effect of varied concentrations of VEGF on the H_2S production in HA-VSMCs. a, HA-VSMCs were incubated with 2.5 μM SF7-AM for 30 min at 37 °C, washed and then imaged using an inverted fluorescence microscope. **b-f**, HA-VSMCs were incubated with varied concentrations of VEGF for 30 min at 37 °C and then imaged. **g-l**, Brightfield images corresponding to **a-f** (Scale bar, 250 μm). **m**, Quantification of the fluorescence images of H_2S signaling in HA-VSMCs, with data from **a-f**. The graph represents the relative fluorescence intensity compared with the relative fluorescence intensity of nontreated cells (**a**) and shows the means ± SD (n=6). An unpaired two-tailed Student's *t* test was used to determine the significance of differences between the two group means. All *p*-values *versus* Control group (nontreated cells).

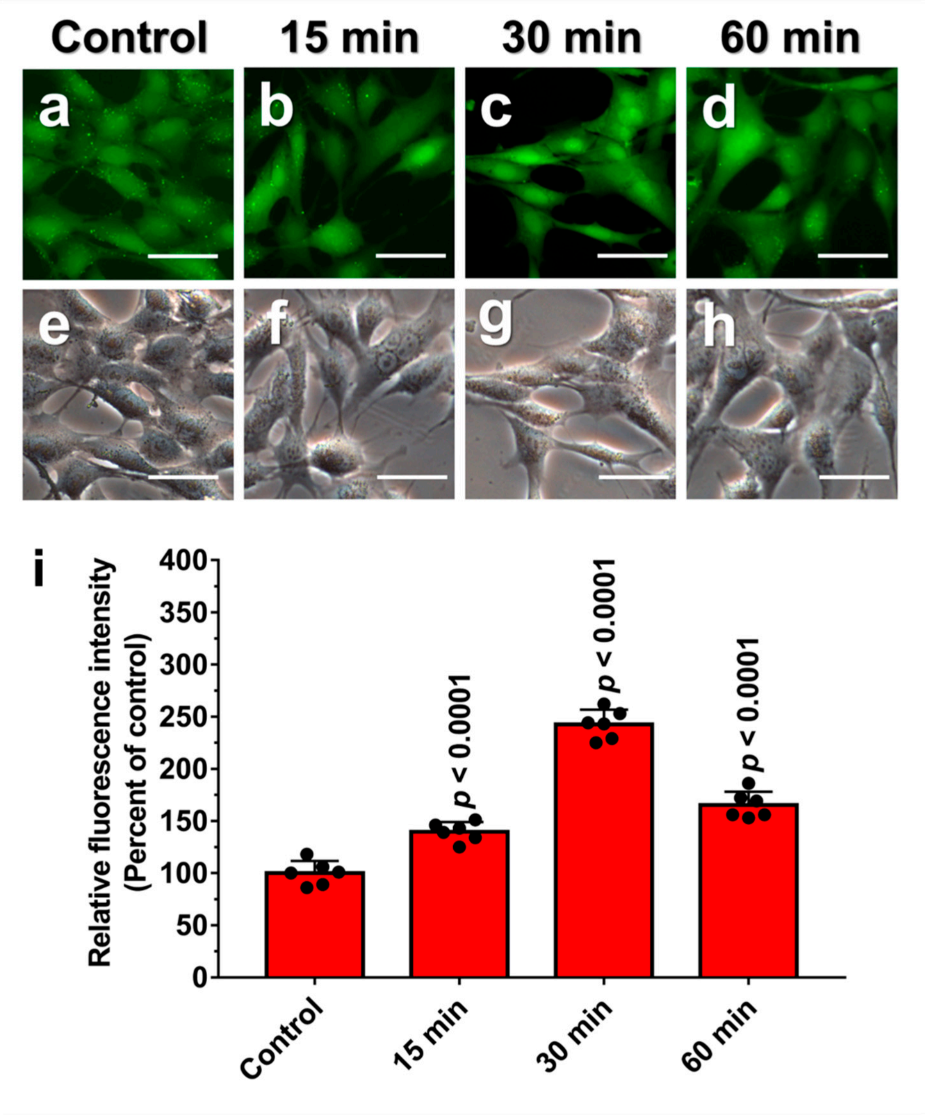

Figure S13. Effect of different times of VEGF (50 ng/ml) treatment on H_2S production in HA-VSMCs. a, HA-VSMCs were incubated with 2.5 µM SF7-AM for 30 min at 37 °C, washed and then imaged using an inverted fluorescence microscope. **b, c** and **d**, HA-VSMCs were incubated with VEGF (50 ng/ml) for different times at 37 °C and then imaged. **e-h**, Brightfield images corresponding to **a-d** (Scale bar, 250 µm). **i**, Quantification of the fluorescence images of H_2S signaling in HA-VSMCs, with data from **a-d**. The graph represents the relative fluorescence intensity compared with the relative fluorescence intensity of nontreated cells (**a**) and shows the means ± SD (n=6). An unpaired two-tailed Student's *t* test was used to determine the significance of differences between two group means. $p < 0.0001$ *versus* Control group.

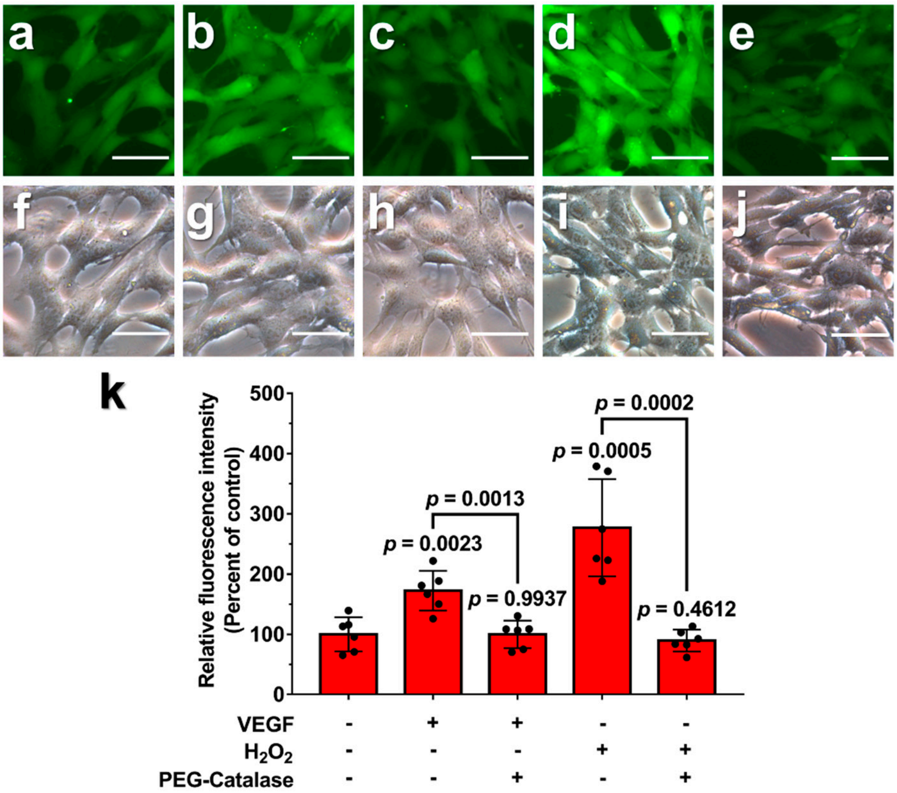

Figure S14. The effect of PEG-catalase treatment on H_2S production in HA-VSMCs. a, HA-VSMCs were incubated with 2.5 µM SF7-AM for 30 min at 37 °C, washed and then imaged using an inverted fluorescence microscope. **b**, Cells were incubated with 50 ng/ml VEGF for 30 min at 37 °C and then imaged. **c**, Cells were incubated with 200 U/ml PEG-catalase (Sigma) for 4 h before VEGF stimulation, followed by the treatment with 50 ng/ml VEGF for 30 min at 37 °C and then imaged. **d**, Cells were incubated with 400 µM H_2O_2 for 30 min at 37 °C and then imaged. **e**, Cells were incubated with 200 U/ml PEG-catalase for 4 h before H_2O_2 stimulation, followed by the treatment with 400 µM H_2O_2 for 30 min at 37 °C and then imaged. **f-j**, Brightfield images corresponding to **a-e** (Scale bar, 250 µm). **k**, Quantification of the fluorescence images of H_2S signaling in HA-VSMCs, with data from **a-e**. The graph represents the relative fluorescence intensity compared with the relative fluorescence intensity of nontreated cells (**a**) and shows the means ± SD (n=6). An unpaired two-tailed Student's t test was used to determine the significance of differences between two group means. *p*-values *versus* nontreated group.

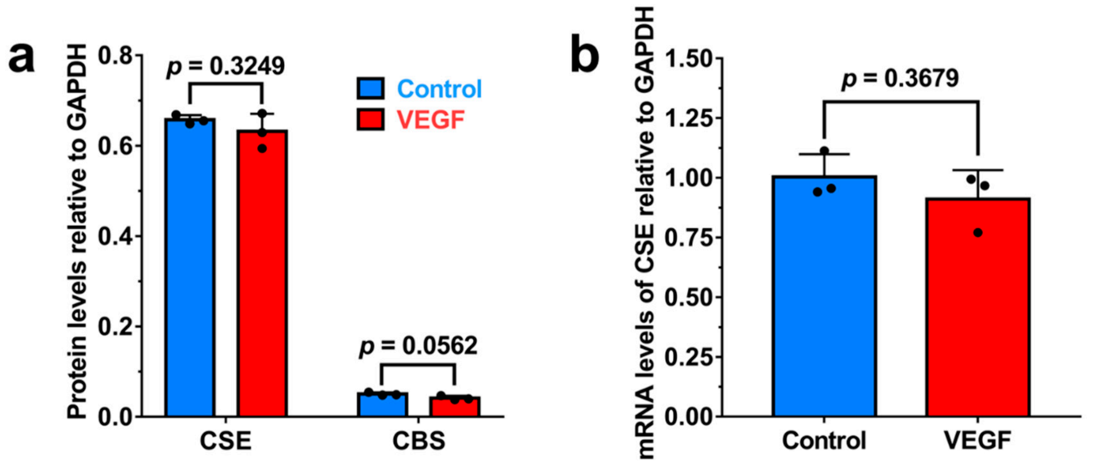

Figure S15. The effect of VEGF on the mRNA and protein levels of CSE in HA-VSMCs. a, HA-VSMCs were cultured to 80% confluence, and then were incubated with 50 ng/ml VEGF for 30 min. Cell lysates were resolved on SDS-PAGE and immunoblotted for CBS, CSE. Glyceraldehyde-3-phosphate dehydrogenase (GAPDH) was used as a loading control. **b,** Cells were incubated with 50 ng/ml VEGF for 30 min, and then the cells were harvested for RNA extraction and cDNA reverse transcription followed by qPCR analysis using SYBR Mix from Agbio (Changsha, China). The level of GAPDH was used as an internal reference gene. The data are presented as the means ± SD (n=3). An unpaired two-tailed Student's *t* test was used to determine the significance of differences between two group means.
